# A Review on *Sarcocornia* Species: Ethnopharmacology, Nutritional Properties, Phytochemistry, Biological Activities and Propagation

**DOI:** 10.3390/foods10112778

**Published:** 2021-11-11

**Authors:** Luísa Custódio, Maria João Rodrigues, Catarina G. Pereira, Viana Castañeda-Loaiza, Eliana Fernandes, Dominic Standing, Amir Neori, Muki Shpigel, Moshe Sagi

**Affiliations:** 1 Centre of Marine Sciences, Campus de Gambelas, University of Algarve, 8005-139 Faro, Portugal; mjrodrigues@ualg.pt (M.J.R.); cagpereira@ualg.pt (C.G.P.); vcloaiza@ualg.pt (V.C.-L.); elianafernandesea@gmail.com (E.F.); 2 French Associates Institute for Agriculture and Biotechnology of Drylands, Blaustein Institutes for Desert Research, Sde Boker Campus, Ben-Gurion University of the Negev, Beersheba 849900, Israel; standing@bgu.ac.il; 3 Morris Kahn Marine Research Station, The Leon H. Charney School of Marine Sciences, University of Haifa, Haifa 3498838, Israel or aneori@gmail.com (A.N.); shpigelm@gmail.com (M.S.); 4 The Interuniversity Institute for Marine Sciences in Eilat, Eilat 8810302, Israel; 5 The Jacob Blaustein Institutes for Desert Research, The Albert Katz Department of Dryland Biotechnologies, Ben-Gurion University, P.O. Box 653, Beer Sheva 84105, Israel; gizi@bgu.ac.il

**Keywords:** glassworts, salt-tolerant plants, salinization, sustainability, halophytes, gourmet foods

## Abstract

*Sarcocornia* A. J. Scott is a halophytic edible succulent plant belonging to the *Amaranthaceae* family. To date, the genus includes 28 species distributed worldwide in saline environments, usually salt marshes. *Sarcocornia* (Scott) is similar to *Salicornia* (L.), which has a recognized commercial value in morphological and taxonomical traits. Species of both genera are commonly named samphire or glassworts in Europe, and their fleshy shoots are commercialized under their traditional names. Due to their nutritional, organoleptic and medicinal properties, *Sarcocornia* species have a high economic potential in various biotechnology sectors. Being highly tolerant to salt, they can be cultivated in saline conditions, and dissimilar to *Salicornia*, they are perennial, i.e., they can be harvested year-round. Therefore, *Sarcocornia* species are considered promising gourmet vegetables to be explored in the context of climate change, soil and water salinization and eco-sustainability. We hereby put together and reviewed the most relevant information on *Sarcocornia* taxonomy, morphology, nutritional and pharmacological properties, uses in ethnomedicine, potential applications in biotechnology, and propagation strategies.

## 1. Introduction

*Sarcocornia* A. J. Scott is one of sixteen genera in the *Salicornioideae* subfamily of the *Amaranthaceae* family [1,2] and includes 28 halophytic species to date, distributed worldwide in saline environments [1,2,3,4,5]. *Sarcocornia* spp. are succulent and edible subshrubs or shrubs, with high phenotypical plasticity, that combined with the reduction in their leaves and flowers, frustrates the unequivocal identification of the species [6]. *Salicornia* (L.) and *Sarcocornia* (Scott) species are morphologically and taxonomically similar and commonly named glassworts [2,5,7]. In Europe, the young fleshy edible tips of *Sarcocornia* and *Salicornia* are commercialized with the name “samphire” or “sea asparagus” [8]. In England, France, the USA, and Australia, they are also known as “herbe de Saint Pierre” (anglicized in the mid-16th century to samphire), “pickleweed”, “poorman’s asparagus” and “sea asparagus” [9]. The succulent shoots are highly valued in gourmet cuisine due to their crunchy texture and salty taste, and have a balanced nutritional profile, in terms of, for example, fiber, and antioxidant vitamins such as vitamin C [10,11,12,13], making them an ideal food supplement [14]. They also contain valuable metabolites, such as polyphenolic compounds, displaying relevant health-promoting properties like antioxidants and anti-inflammatory [15]. Additionally, *Sarcocornia* is perennial and can be cultivated under saline conditions, which is highly relevant in the context of soil and water salinization and the scarcity of fresh water for agriculture. However, information related to *Sarcocornia* spp. is widely scattered throughout the literature. This review provides a detailed overview of the botanical aspects, ethnomedicinal uses, economic aspects and phytoconstituents of *Sarcocornia* species along with its nutritional and functional properties, with particular emphasis on human health improvement. Finally, an insight into *Sarcocornia* propagation methods is given.

## 2. Methodology

We consulted the database of PubMed, Web of Science, Embase, and Google Scholar (as a search engine) to retrieve the most updated articles. The keyword “*sarcocornia*” was used, alone or in combination with, for example, “ethnopharmacology”, “cultivation”, “propagation”, “in vitro culture techniques”, “micropropagation”, “nutritional, “bioactivities”, “antioxidant”, “anti-inflammatory” and “anticancer”. Only English articles with a full text were considered.

## 3. Botanical Aspects

**Figure 1:**
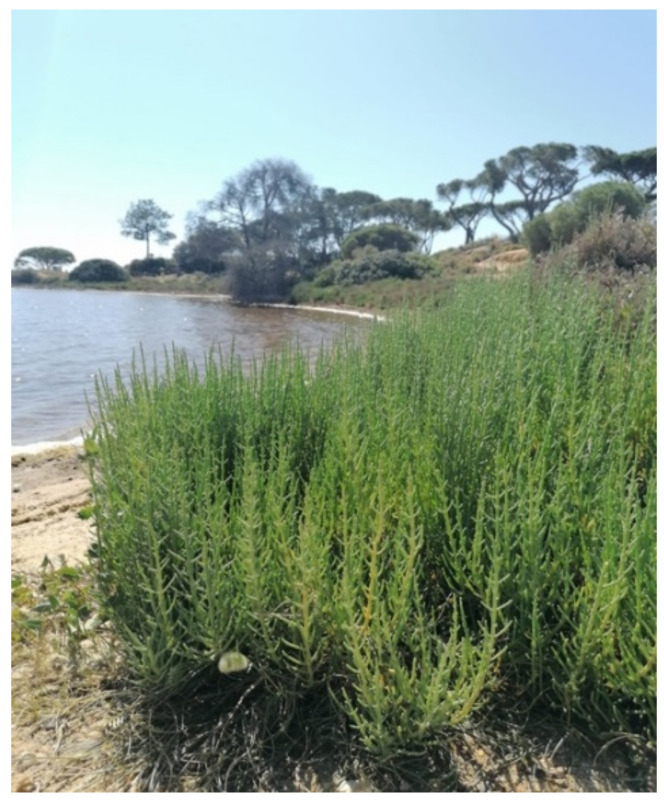
*Sarcocornia fruticosa* in a salt marsh of the Southern Algarve, Portugal. Photo by Maria J. Rodrigues.

The *Sarcocornia* genus, described by A.J. Scott in 1978, includes, to date, 28 perennial species [2] formed by small bushes, erected or prostrate, highly branched, and up to 150 cm tall (Figure 1). These plants have woody stems in the basal part, with the upper parts being fleshy and jointed with opposite leaves. They present opposite bracts with a spiciform inflorescence with the ops of three flowers in the armpit of each bract. Flowers are hermaphroditic and seeds are ellipsoid, small (0.10 ± 0.04 mg), develop inside fertile segments of succulent green shoots, and are only available after the senescence of shoots [16]. The genus *Sarcocornia* is now distinguished from the annual *Salicornia* by its different perennial growth pattern [4] and differences in the flower arrangement [17]. *Sarcocornia* species can grow under salt concentrations up to 1030 mM NaCl and prolonged flooding periods, and are distributed worldwide in coastal salt marshes, tidal mud flats, coastal cliffs, inland salt pans, edges of saline lakes and saline deserts [1,2,8]. They are rarely found in low salinity areas, except for *Sarcocornia xerophila* (Toelken) A.J. Scott in South Africa [3].

Halophytes are exposed to environmental stress, including hypersalinity, which produces an ionic imbalance and hyperosmotic stress that negatively affect plant growth, seed germination, seedling growth and vigor, vegetative growth, flowering, and fruitification [18]. Hypersaline stress triggers the production of reactive oxygen species (ROS) within cells [18]. As defense mechanisms against salt stress and ROS production, plants develop, in general terms, anatomical (such as salt glands, salt bladders and succulence; e.g., *Salicornia dolichostachya* Moss) and biochemical strategies (including the synthesis of compatible solutes, and antioxidant enzymes, selective accumulation or exclusion of specific ions, compartmentalization of ions at the cellular- and whole-plant levels; e.g., *Salvadora persica* L., *Centaurea tuzgoluensis* Aytaç & H. Duman) [19,20,21]. The mechanism of salt tolerance of *Sarcocornia* species is based on succulence (increased water content in shoots), elimination of excess Na^+^ and Cl^−^ with the detachment of old leaves, ability to maintain a lower K^+^/Na^+^ ratio and higher organ selectivity of K-Na [22].

## 4. Ethnomedicinal and Biotechnological Uses

Glassworts produce succulent shoots with a very much appreciated salty taste. Therefore, its most common traditional use is as food, either raw (e.g., in salads) or cooked (similar to spinach), especially as side dishes with fish or seafood or dried as a salt substitute [23,24]. *Sarcocornia* species have ethnomedicinal uses for their diuretic and depurative properties [23,25]. They also contain bioactive ingredients with great potential for the pharma industry, including polyphenols, flavonoids, and vitamins [10,11,12,13]. Seeds of *S. ambigua* (Michx.) M.A. Alonso & M.B. Crespo have a high fatty acid content and potential for animal consumption and/or biofuel production [26]. *Sarcocornia fruticosa* (L.) A.J.Scott can produce a high amount of biomass with suitable lignocellulosic materials adequate for bioethanol production [27]. At the same time, *S. perennis* can be used to phytoremediate metals in polluted soils due to its high capacity to accumulate ions and cope with oxidative stress related to metal accumulation in plant tissues [28].

## 5. Economic Importance

There is little information available on wholesale prices of *Sarcocornia* [29], or regarding the evaluation of the economic viability of its production, even though it is currently marketed in countries worldwide as a fresh vegetable in elite kitchens due to its taste and medicinal properties [12,13,30]. For example, Castillo-Barros et al. [31] made a commercial-scale economic evaluation on the cultivation of *S. ambigua* combined with the production of the shrimp *Litopenaeus vannamei* in a marine aquaponics production system located on the coast of Southern Brazil. A cash flow horizon of 10 years was used to calculate the total cost of production (TCP) and the financial viability of the process. The predicted value for the initial investment was US$474,253.07, the estimated annual TCP values ranged between US$192,220.05 and US$247,740.52, and the annual production of *Sarcocornia* varied between 17,017 and 23,286 Kg. Based on these annual production numbers, the predicted TCP was approximately US$4. A total of 3 sales prices were used per kilogram of *Sarcocornia* produced (US$9.00, US$10.00, and US$11.00), and only the highest price allowed for satisfactory indexes after testing sensitivities. The authors concluded that the *Sarcocornia* TCP was below the reference price and could therefore be increased to reach a “premium” level, and that the analyzed production system was feasible for implementation in Brazil. In another study in the Southern Brazil, the economic viability of the production of *S. ambigua* in a hydroponic system over ten years was also confirmed, with the following proposed values: initial investment, US$15,529.21; total operation cost (TOC), US$20,294.76–US$20,525.01; production per 203 m^2^ per week, 35 Kg; selling price: US$19.72/Kg [32]. The retail value of *Sarcocornia* in Europe can be estimated by the current values for *Salicornia* (identical to *Sarcocornia*, previously known as perennial *Salicornia*), at around 12 €/Kg; some European supermarkets also maintain online groceries listings where current prices can be consulted. In Portuguese markets, *Sarcocornia* spp. are sold as sea asparagus along with *Salicornia* spp. Although no data are available on its global market size, the growing public interest is reflected in the increasing number of brands selling it [33]. Overall, the growth of *Sarcocornia* remains a niche and has excellent potential for development [8,12,24].

## 6. Nutritional Properties

The following sections contain information on the most relevant nutritional properties of different *Sarcocornia* species in the context of human nutrition. Whenever applicable, data is compared with *Salicornia* and other worldwide consumed green vegetables, such as lettuce (*Lactuca sativa* L.).

### 6.1. Proximate Composition

The proximate composition of some *Sarcocornia* species is presented in Table 1. *Sarcocornia* species are characterized by a high moisture content, generally above 80%, as observed in *S. perennis perennis* (85.8%) and *S. perennis alpini* (84%) collected from the wild [13], *S. ambigua* (approximately 88%) [34] and *S. neii* (86%–89%) [35]. Wild *S. fruticosa* collected in Spain and Portugal also had high moisture contents, especially the cultivated plants (92%) [36]. Such moisture levels are in line with those reported for *Salicornia* species, including *S. ramosissima* [13,37,38], *S. bigelovii* [39] and lettuce [40]. The moisture level impacts shelf life and the consumption quality of fresh vegetables and may also influence food safety [41]. For example, lettuce leaves with higher moisture content suffered a more pronounced weight loss during storage and were more prone to spoilage [42]. Fresh vegetables with a longer shelf life can conserve a fresh appearance for longer periods [43], significantly impacting the consumers’ acceptance of a product. Higher consumer rejection was related to products with reduced shelf life [44]. Therefore, *Sarcocornia* species with a higher moisture content may lead to a greater risk of lower consumer acceptability.

In general, halophytes have a higher ash level than other edible plants [45], which may be explained by the saline environment in which they grow and their ability to retain minerals [46]. In *S. perennis perennis* and *S. perennis alpini* collected in Southern Portugal, the ash levels varied between 23.3 and 30.7% (dry weight, dw), similarly to the values detected in *S. ambigua* collected in two different locations in Santa Catarina (Brazil) (24.9–31.8%, dw) [34]. *Sarcocornia neii* had higher ash levels, around 35% for wild and cultivated plants [35]. The ash levels of *S. fruticosa* were also high, ranging from 28.9% (dw) in wild plants collected in Spain to 43.3% (dw) in cultivated plants [36]. The *Sarcocornia* ash levels are similar to those observed for *S. ramosissima* collected in Southern Portugal (29.2% dw) [13], but lower than the same species sampled in Aveiro (Portugal) (41–44.2%, dw) [38], which may be related with the mineral composition of the soils from which the plants were collected. In general, the ash levels of *Sarcocornia* were higher than those reported for lettuce grown in both hydroponics (1.18%, dw) and soil (1.32%, dw) [47].

Halophytes also tend to be rich in dietary fibre [46], whose consumption is linked to important health benefits including weight loss due to its low caloric content, and a reduced risk of cardiovascular diseases and diabetes [48,49]. *Sarcocornia perennis perennis* and *S. perennis alpini* presented high levels of neutral detergent fibre (NDF), from which the amounts of fibres, such as cellulose, hemicellulose and lignin as well as cutin and tannins, can be estimated to appraise the insoluble portion of dietary fibre in food [50]. NDF values were 20.8% (dw) for *S. perennis perennis* and 34.1% (dw) for *S. perennis alpini* (dw), respectively [13], and were higher than those of most vegetables, including lettuce [50], and *S. ramossima* [13]. It was suggested that the intake of 100 g of edible portions (fresh weight, fw) of *S. perennis alpini* or *S. perennis perennis* could cover 48% and 71%, respectively, of the recommended daily fiber dose in adults [13]. In *S. ambigua* the insoluble fiber levels fluctuated between 17.2 and 23.2%, depending on the collection site [34]. The crude fiber contents for *S. neii* was 9.98 and 16.9% (dw) for wild and cultivated plants, respectively [35]. The total dietary fiber levels were high in wild plants collected in Spain (22.0%, dw) and Portugal (16.6%, dw), but significantly lower in cultivated plants (9.26%, dw) [36].

Similarly to most vegetables, *Sarcocornia* species are low in protein. *Sarcocornia perennis alpini* and *S. perennis perennis* presented a low protein content (8.10% and 6.90%, dw), similar to *S. neii*, which was higher in the wild (13.5%, dw) than in cultivated plants (6.98%, dw) [35]. The opposite trend was observed in *S. fruticosa*, where the crude protein was higher in cultivated plants (12.6%, dw) when compared to wild plants from Spain and Portugal (about 9%, dw) [36]. Higher protein content was found in *S. ambigua *(16.2–18.02%, dw) [34]. Protein synthesis can be boosted as a result of stress response, triggering short (e.g., modifications in osmotic potential and salt ion activity) and long-term modifications (e.g., structural adaptations of plant cells causing alterations in plant growth and development) [51]. Wild plants are exposed to natural stress conditions, including fluctuations in temperature, salinity, and UV radiation, compared to cultivated ones grown in controlled conditions, which may explain their higher protein content [35]. The total protein levels of *Sarcocornia* are generally in the range, or higher, of those found in *S. ramosissima* collected in Southern Portugal (5.20%, dw) [13] and Aveiro (8.48–13.4%, dw) [38], and much higher than the reported average contents of green leaf lettuce (1.4%, fw) [52].

Overweight/obesity is a significant public health concern worldwide and a risk factor for several health problems, such as hypertension, cardiovascular diseases, and diabetes [53]. Consuming the low-fat Sarcocornia can contribute to weight loss. The fat contents of shoots from *S. perennis perennis* and *S. perennis alpini* collected in Southern Portugal was 2.25 and 1.20% g (dw), respectively [13]. The fat content in *S. ambigua* collected in Brazil was also low (0.12–0.16%, fw) [34]. Although low, the fat level of *Sarcocornia* is usually higher than that of *Salicornia* and lettuce [13,52].

### 6.2. Minerals

Humans need various dietary minerals for health and metabolism since they have pivotal roles in the human body, such as bone formation and muscle function [54]. Minerals are divided into macro-minerals (calcium (Ca), magnesium (Mg), potassium (K), sodium (Na), chloride (Cl), phosphorus (P) and sulfur (S)) and micro-minerals (iodine (I), zinc (Zn), selenium (Se), iron (Fe), manganese (Mn), copper (Cu), cobalt (Co), molybdenum (Mo), fluoride (F), chromium (Cr) and boron (B)), and are present in different food sources, including vegetables, fruits and animal products.

Halophytes can be valuable sources of essential minerals, and the mineral composition of some *Sarcocornia* species is presented in Table 2. Sodium is an essential nutrient, vital in maintaining electrolyte and fluid balance, heart function, muscle contraction and nerve transmission [54]. However, its excessive consumption is linked to several pathologies, including hypertension and cardiovascular diseases [55]. Sodium was the prevalent mineral in *S. perennis perennis* and *S. perennis alpini* shoots collected in Southern Portugal (approximately 64 mg/g, dw) along with K, Mg, Ca and Fe [13]. The Na levels of those *Sarcocornia* species were lower than that detected in *S. ramosissima* (89.9 mg/g) collected in the same location [13] and in Aveiro, Portugal (114–147 mg/g) [38]. Antunes et al. [56] also considered Na the primary mineral (16%) in wild *S. perennis perennis* from Southern Portugal. Sodium was also the primary mineral in shoots of wild *S. fruticosa* collected in Cádiz (Spain) (243 mg/g, dw), in Ria Formosa Natural Park (Portugal), (253 mg/g, dw), and in cultivated plants (297 mg/g, dw) [36]. These levels were higher than in *Sarcocornia* and *Salicornia* from Southern Portugal [13]. Shoots from *S. ambigua* from two populations in Brazil had Na levels ranging from 10.19 mg/g to16.57 mg/g (fw), followed by P, Mg and Ca [34]. The Na content in the referred *Sarcocornia* species was higher than in common green vegetables, including lettuce (0.8–44 mg/g, dw) [40] and seaweed (1.46–39.6 mg/g, dw) [57]. The World Health Organization (WHO) recommends that the Na daily intake should not exceed 2000 mg. Consuming 100 g of fresh shoots of *S. perennis alpini* and *S. perennis perennis* would represent intakes of 1029 and 910 mg of Na, respectively. Hence, caution must be taken so that the maximum allowed daily dose recommended by the WHO is not exceeded.

Potassium, an essential nutrient for nerve transmission, muscle contraction and adequate fluid balance [54], was the primary macroelement in shoots from wild (2.03 g/100 g, fw) and cultivated (1.55 g/100 g, fw) *S. neei* plants [35], and the second most abundant mineral in *S. perennis alpini* (10.3 mg/g, dw) and *S. perennis perennis* (13.9 mg/g, dw), after Na [35]. The K content in *Sarcocornia* is higher than in *S. ramosissima* [13,38], different lettuce types (1.4–6.5 mg/g, fw) [40], and spinach, which is considered a good vegetable source of K [52].

Iron is a crucial component in haemoglobin and in energy metabolism, and integrates important enzyme systems [54]. Iron and Zn are of particular concern for vegan diets since these elements are generally present in low amounts in plant products [40]. Approximately 2 billion people are anaemic and of them approximately 50% are Fe-deficient [58]. Iron was a prevalent micronutrient in wild and cultivated *Sarcocornia* species (0.33–1.69 mg/g, dw) [13,35,36,56], and in *S. ramossima* collected in Portugal, both in Aveiro (0.045–0.073 mg/g, dw) [38] and in the Algarve (1.53 mg/g, dw) [13]. The Fe content in *Sarcocornia* is also generally higher than in lettuce (0.0599–0.248 mg/g, dw) and in Fe rich vegetables, such as parsley (*Petroselinum crispum* (Mill.) Fuss; 0.062 mg/g) [52]. Therefore, only 20 and 45 g of fresh *Sarcocornia* could cover the recommended daily Fe intake of 8–18 mg/day for adults [59]. However, it must be highlighted that the Fe form provided by vegetables (non-heme) is less bioavailable than that provided by animal products (heme) [40].

Bertin et al. [60] evaluated the mineral levels and mineral bioaccessibility of *S. ambigua* shoots from wild and irrigated populations, in Brazil, after in vitro gastric and intestinal digestion. The prevailing minerals were K (19–24 mg/g), Mg (8.6–14 mg/g) and Ca (2.6–4.0 mg/g). A high fraction (65–80%) of K and Mg (80 and 65%) was bioaccessible. In addition, more than 50% of the total concentration of the trace elements vanadium (V), Cr, cobalt (Co), Cu and lithium (Li) was also bioaccessible. The authors suggested that the consumption of 2 g of *S. ambigua* shoots provides a small part of the recommended daily intake of K, Ca, and Zn, but a higher level of Mg, Mn, Cu, and Cr [60]. Likely, the ingestion of 2 g of *S. ambigua* per day would not cause adverse health effects since bioaccessibility studies suggest that the recommended dietary allowance (RDA) values are not exceeded for any of the elements [60].

### 6.3. Fatty Acids (FA)

FA are vital energy sources and cellular membranes constituents. They can be divided according to the number of double bonds present in their carbon chain into saturated (SFA), monounsaturated (MUFA) and polyunsaturated (PUFA). The FA composition of food dramatically impacts its quality and may influence the overall consumer health. As vertebrates are unable to synthesize α-linolenic (C18:3n-3; ALA) and linoleic (C18:2n-6; LA) acids, these PUFAs must be obtained through diet and are thus among the most important required nutrients in both food and feed [61].

The FA composition of some *Sarcocornia* species is presented in Table 3. The crude fat fraction in *Sarcocornia perennis perennis* and S. *perennis alpini* collected in Southern Portugal contained a rather saturated FA methyl esters (FAME) profile, with the sum of SFA always higher than 50%, and low levels of MUFAs, namely 10.7% for *S. perennis alpini* and 2.47% for *S. perennis perennis* [13]. Such FA saturation profiles are typical in halophytes and are probably related to salt tolerance mechanisms, such as decreased membrane permeability to sodium chloride (NaCl) [62]. In the studied *Sarcocornia* species, the PUFA fraction represented 36.9% (*S. perennis alpini*) and 39.9% (*S. perennis perennis*) of the total FA and was dominated by α-linolenic acid (ALA; 18:3n-3) and linolenic acid (LA) [13]. ALA is a precursor of eicosapentaenoic (EPA, 20:5(n-3) and docosahexaenoic acids (DHA, 22:6(n-3). Its consumption is associated with higher ALA, EPA, docosapentaenoic acid (DPA; 22:5n-3) and, to a lesser extent, DHA tissue contents. Alpha-linolenic acid may reduce arachidonic acid (ARA; 20:4n-6) levels by competing with LA for the same metabolic enzymes (*e.g.*, responsible for the elongation of n-6 FA), reduces the levels of serum triglycerides and displays anti-inflammatory properties, which may help in the prevention of cardiovascular diseases and has a positive effect on the recovery of central nervous system injuries [63]. A predominance of ALA and LA was also observed in other vegetables, including lettuce [64]. In general, the FA profiles of the reported *Sarcocornia* species are similar to those described for *Salicornia* [12,13,65,66].

Barreira et al. [13] determined the indexes of atherogenicity (IA) and thrombogenicity (IT), which are commonly used to predict the health benefits associated with the consumption of several foods. The IA indicates the relationship between the main classes of saturated and unsaturated FA. Since saturated FA are proatherogenic and unsaturated FA anti-atherogenic, a low IA ratio is desirable [67]. The IA values for *S. perennis alpini* and *S. perennis perennis* were 0.62 and 0.55, respectively, lower than in *S. ramossima* collected in the same location [13]. The IT indicates the tendency to form clots in the blood vessels and is defined as the link between the pro-thrombogenic (saturated FA) and the antithrombogenic (MUFA, n-3 and n-6 PUFA). Therefore, a low value of IT is also desirable [67]. The IT values for *S. perennis alpini* and *S. perennis perennis* were 0.37 and 0.28 [13], similar to the IT for *S. ramossima*, and also to those calculated for tuna fish, which is considered a “healthy” supply of unsaturated FA [13,67]. The PUFA/SFA ratios in *S. perennis alpini*, *S. perennis perennis* and *S. ramossima* were above 0.45, considered beneficial for good nutritional quality and health [13]. The FAMES in whole plants from *S. ambigua* collected in different Brazilian areas had a predominance of PUFAs, approximately 60%, mainly linolenic and linoleic acids. In comparison, SFA represented circa 17% of the total lipids content, mainly palmitic acid, and MUFAs represented approximately 4%, primarily oleic acid [34].

The fat levels in *Sarcocornia* are low in the aerial vegetative organs and high in seeds, and therefore, these species are considered potential seed-oil crops [16]. The FAME content of the seed oil from *S. ambigua* had a higher level of PUFAs (47.5%) than SFAs (24.9%) and MUFAs (20.5%) [26]. Among the detected PUFAs, LA (C18:2n-6), which is an essential fatty acid in human nutrition when consumed in moderate amounts [68], was the most abundant (42%) [26]. During harvesting, seeds are typically mixed with debris such as soil and remnant cellulose material, which are difficult to separate, and this mixture is designated as “meal”. SFAs were dominant in the meal of *S. ambigua*, especially palmitic acid (C16:0, 19.8%), stearic acid (C18:0) and myristic acid (C14:0) [16]. Part of this high SFA content may be due to the presence of seeds in the meal [16,26].

### 6.4. Vitamins

Vitamins are essential for the adequate growth and functioning of the human body that can only be acquired from the diet. They are divided into water-soluble and fat-soluble vitamins. Tocopherols (α, β, γ, and δ forms) are fat-soluble compounds with vitamin E activity, with recognized antioxidant properties [69]. Alpha tocopherol is the most potent and bioactive form of vitamin E and can be obtained from different plant sources, especially oilseeds [70]. The vitamins content of some *Sarcocornia* species is summarized in Table 4.

*Sarcocornia perennis perennis* and *S. perennis alpini* collected in Southern Portugal had α-tocopherol levels of 0.0111 and 0.0201 mg/g (dw), respectively, the latter being significantly higher than in *S. ramosissima* [13]. However, the γ-tocopherol levels in such *Sarcocornia* species were significantly lower: 0.0009 in *S*. *perennis perennis* and 0.0096 mg/g in *S. perennis alpini*, but undetected in *S. ramossima * [13]. In *S. fruticosa*, vitamin E was only detected in plants collected from the wild, both in Spain (0.176 g mg/g, dw) and Portugal (0.934 mg/g, dw). The α- and γ- tocopherol contents of *Sarcocornia* species were within the range reported for different lettuce types (0.022–0.148 mg/100 g, dw) [40].

Ascorbic acid (vitamin C), which is known for its capacity to prevent scurvy [71], was detected in shoots from *S. ambigua* cultivated using effluents from shrimp culture. Levels were especially high in the vegetative segments, with values ranging from 0.237 mg/g (fw) in green reproductive to 0.654 mg/g (fw) in red vegetative tissues [72]. Vitamin C was also detected at similar levels in the wild (0.55 mg/g, dw) and cultivated plants (0.9 mg/g, dw) of *S. neei* [35] and in the wild and cultivated plants of *S. fruticosa* from Spain and Portugal (0.0038–0.0076 mg/g, dw) [36]. Thus, the vitamin C content in *Sarcocornia* species is in the range, or even higher, than the average vitamin C level in lettuce (0.0046 mg/g, fw) [52].

Vitamin B6 (pyridoxine) is vital for general cellular metabolism, has a role in chlorophyll synthesis, exhibits antioxidant properties, and is therefore implicated in the resistance to abiotic and biotic stress [73,74]. Vitamin B6 was detected in cultivated and wild plants of *S. fruticosa* (0.0667–0.0981 mg/g, dw) [36] in higher levels than in lettuce (0.001 mg/g, fw) and in some vegetables considered as good sources of vitamin B6, such as dried pasilla peppers (*Capsicum annuum* L.; 0.042 mg/g, dw) [52].

## 7. Bioactive Components

Halophytes, including *Sarcocornia* species, contain primary and secondary metabolites (e.g., vitamins, minerals, phenolics) with pivotal plant protective roles and important functional properties (e.g., antioxidant, anti-inflammatory). These metabolites have several biotechnological and commercial applications in the food and pharmaceutical industries and elsewhere [8,13,65,75,76,77,78,79,80,81].

### 7.1. Polyphenolic Compounds

Polyphenolic compounds are secondary metabolites ubiquitous in plants that can exhibit important biological properties potentially associated with multiple health benefits [82]. Halophytes, including *Sarcocornia* species, usually have a high phenolics content as part of their antioxidant defense mechanism (Table 5). A methanol extract of *S*. *perennis*’s aerial parts collected in Tunisia contained 4.32 mg of gallic acid equivalents (GAE)/g (dw) of total phenolic compounds (TPC) and 3.3 mg of catechin equivalents (CE)/g (dw) of total flavonoids (TFC) [83]. Shoots from *S. perennis alpini* and *S. perennis perennis* collected in Southern Portugal contained much TPC (20.7 and 20.5 mg GAE/g, dw) and TFC (14.3 and 19.9 mg CE/g, dw) [13]. The TPC levels in two *Sarcocornia* species were lower than in *Salicornia ramossima* (33 mg GAE/g, dw), collected from the same area, while the levels of flavonoids were similar [13]. Shoots from *S. perennis* collected in Southern Portugal also had a high content of total phenolics, which increased after 14 days of storage at 4 °C when compared with biomass immediately after harvest, in May and July (at harvest, in May: 32.74 mg GAE/g (dw), after 14 days: 33.84 mg GAE/g (dw); at harvest, in July: 25.58 mg GAE/1 g (dw), after 14 days: 35.61 mg GAE/g (dw), determined by the Folin-Ciocalteu assay) [56]. This was attributed to postharvest plant stress, which stimulates the biosynthesis of polyphenols [56]. The same authors also observed a decrease in the total phenols (13.19 to 10.60 mg/g), hydroxycinnamic acid derivatives (3.66 to 1.20 mg/g), and flavonoids (3.05 to 2.11 mg/g) from May to July harvests. The higher exposure of plants to UV radiation occurring in July may be responsible for the decrease in phenolic levels since at these conditions the role of such compounds in the defense against damaging UV-radiation is less important [56]. A decrease in the levels of phenolic compounds in *Salicornia ramosíssima* shoots after UV treatments was also observed, associated with a putative down-regulation of phenol synthesis to reallocate energy towards other photoprotective pathways able to slow down chlorophyll degradation, including the synthesis of carotenoids [84]. It is accepted that natural extracts are rich in phenolics when GAE values are higher than 20 mg/g dw [85], therefore, data shows that *Sarcocornia* species are in general good sources of such compounds.

Bertin et al. [34] identified fifteen phenolic compounds in shoots from wild and cultivated *S. ambigua* (Brazil) by high-performance liquid chromatography/electrospray ionization tandem mass spectrometry (HPLC–ESI-MS/MS). Wild plants had higher diversity and content of flavonoids and phenolic acids than cultivated ones, and the major phenolics identified were ferulic acid, caffeic acid and galangin [34] (Table 5). The phenolic compounds were determined in different extracts (water, 80% aqueous ethanol, 80% acidified aqueous methanol and a mixture of methanol/ethanol/water (40/40/20)) of fresh vegetative and reproductive segments (with seeds) of green, pink, and red *S. ambigua* biotypes, cultivated under irrigation with saline effluent from shrimp production tanks, in Brazil [72].

The highest level of individual contents of phenolics was detected in the reproductive segments of the red biotype of *S. ambigua* (186–365 mg/100 g, fw). The major phenolics were flavonoids, mainly kaempferol and quercetin, followed by gallic acid and other hydroxybenzoic acids, present in higher levels in reproductive segments of the red biotype (Table 5) [72]. The red biotype is predominant in stressful habitats of salt marshes. Its higher phenolics content may be associated with a higher tolerance to high salt concentration, light exposure and/or temperature fluctuations [86]. Castañeda-Loaiza et al. [36] compared methanol extracts made from dried biomass of *S. fruticosa* collected between March and May 2018 from wild populations in Portugal and Spain with those cultivated in greenhouse conditions. Plants collected in Spain showed the highest content of total phenolics and flavonoids, which differed in their variety from the other sources. Generally, cultivated plants had the highest phenolics content; the main compounds detected were chlorogenic acid, catechin hydrate and 3,4-dihydroxybenzoic acid (Table 5). The content of phenols and flavonoids can vary between species, ecotypes [8,12,87], and the extraction procedure. A total of 5 flavonols were isolated and identified from a 70% methanol extract from *S. fruticosa* leaves collected in Saudi Arabia, namely rhamnazin 3-*O*-2G-rhamnorutinoside or rhamnazin 3-*O*-(2′′,6′′-*O*-α-di-rhamnosyl)-β-glucoside, rhamnazin 3-*O*-rutinoside, rhamnazin 3-O-(6′′-*O*-α-rhamnosyl)-β-galactoside, isorhamnetin 3-*O*-(6′′-*O*-α-rhamnosyl)-β-galactoside, isorhamnetin 3-*O*-(2′′,6′′-*O*-α-di-rhamnosyl)-β-galactoside and allantoin (Table 5) [88]. Except for allantoin, all of the compounds are flavonoid-3-*O*-glycosides, i.e., phenolic compounds containing a flavonoid moiety which is *O*-glycosidically linked to carbohydrate moiety at the C3-position [89], and display important bioactivities, such as antioxidant [90]. Allantoin is a nitrogen-containing metabolite and an intermediate product of purine metabolism [91], accumulates in high levels under various stress conditions, including excessive UVA radiation and hypersalinity [92], and has emerged as a molecule involved in increasing stress tolerance in plants [93,94]. It was identified in different halophytes such as *Crithmum maritimum* L [7] and *Suaeda fruticosa* (L.) Forssk. [95] and is an active cosmetic ingredient with moisturizing and re-epithelization properties [96].

### 7.2. Polysaccharides

Plant cell wall polysaccharides display several important health-promoting properties, including immunomodulation and antitumoral [97,98]. Several polysaccharides were identified in an 95% ethanol extract from vegetative organs of *S. perennis perennis* collected in Portugal, and consisted mainly of uronic acids, glucose and arabinose, and also rhamnose, fructose, xylose, mannose, and galactose [99]. The biological properties of such compounds are discussed in Section 8.

## 8. Biological Properties

### 8.1. Antioxidant Activity

Halophytes contain effective enzymatic and non-enzymatic antioxidant systems, which help them cope with oxidative stress in the harsh environment where they thrive. As a result, halophyte’s extracts, including from *Sarcocornia* species, exhibit antioxidant properties (Table 6). Water, 80% ethanol, 80% acidified aqueous methanol and methanol/ethanol/water (40/40/20) extracts of vegetative and reproductive segments of *S. ambigua*’s biotypes displayed in vitro antioxidant properties in the 2,2-azinobis-(3-ethylbenzothiazoline-6-sulfonic acid) (ABTS) assay (3.4–4.9 mmol Trolox equivalent antioxidant capacity (TEAC)/100 g, fw). This activity was particularly pronounced in the reproductive tissues and was positively correlated with the extracts' gallic acid and total phenolic acids contents [72]. Shoot ethanol extracts of *S. perennis alpini* and *S. perennis perennis* collected in Southern Portugal displayed also radical scavenging properties towards the 2,2-diphenyl-1-picrylhydrazyl (DPPH) radical (IC_50_ values of 11.5 and 8.04 mg/mL, respectively), coupled with iron reducing properties (IC_50_ values of 6.55 and 4.57 mg/mL, respectively) [13]. A methanol extract of *S. perennis* aerial parts collected in Tunisia could scavenge DPPH radicals (IC_50_ = 0.43 mg/mL) linked to its phenolic contents [15]. Methanol extracts from *S. ambigua* collected from a natural tideland (Region A) and from cultivated plants irrigated with seawater and fertilized with sludge from shrimp productions tanks (Region B) in Brazil showed significant antioxidant activity, especially those from region A (135.83 μmol TEAC/100 g) [34]. This is most probably due to the higher salt concentrations in the soil of region A, that induces ROS production and oxidative damage and a consequent higher synthesis of antioxidants [34,100]. The flavonols isorhamnetin di- and tri-glycosides isolated from an aqueous methanol extract of *S. fruticosa* leaves from Saudi Arabia were effective DPPH scavengers, with IC_50_ values of 3.8 and 4.3 μM [88]. Antunes et al. [56] prepared ethanol/water (75:25) extracts from *S. perennis shoots* collected from wild populations in Southern Portugal in May and July and evaluated them for antioxidant activity, by the TEAC and oxygen radical absorbance capacity (ORAC) methods, at harvest and after 14 days of storage at 4 °C. Samples collected in May and after 14 days postharvest had a higher antioxidant activity, with values of 2.54 mM Trolox/100 g and 8.84 mM Trolox/100 g, for TEAC and ORAC, respectively, probably due to the higher levels of phenols [56]. 

Lead (Pb) is one of the most prevalent environmental toxins whose levels have increased more than 100-fold over the past three centuries, mainly due to human activity [83]. Pb induces oxidative stress in cell cultures. In animals, Pb causes, for example, hepato- and nephro-toxicity [83,101,102,103,104,105,106,107,108]. Incubation of HEK 293 kidney cells with Pb for 24 h resulted in cytotoxicity and oxidative damage, as denoted by morphological changes, including cell rounding, continuous cell detachment and increased number of non-adherent cells, and increased release of superoxide anions (O_2_^·−^) [15]. The simultaneous application of Pb and a methanol extract from *S. perennis* aerial organs prevented the cellular morphological changes described previously, and O_2_^·−^ release [15]. The methanol extract was then fractionated on reverse phase silica gel with increasing methanol solutions (20 to 100%). The application of the methanol fraction at 80%, and to a lesser extent, at 60%, reduced apoptosis, morphological modifications, O_2_^·−^ production and lipid peroxidation, thus suggesting that the extracts contained compounds capable of counteracting Pb intoxication [15]. A *S. perennis* water extract was further tested against Pb toxicity in rats by treating the animals with the extract (mixed with food), Pb (mixed with the drinking water at 6 g/L) or a combination of both [83]. Treatment with Pb induced oxidative stress as expressed by a significant increase in inflammatory cytokines and of thiobarbituric acid reactive substances (TBARS) and decrease in the levels of interleukin 10 and glutathione (GSH), and in the activities of the antioxidant enzymes superoxide dismutase (SOD), catalase (CAT) and glutathione-peroxidase (GPx) [83]. Treatment with the extract significantly increased antioxidant enzyme activities after 3 weeks, without toxic effects, suggesting that it could be explored as a food supplement to protect against Pb toxic effects [83]. The protective action of the extract could be related to the presence of molecules that may act as ROS scavengers, such as flavonoids [15].

Pectic polysaccharides from *S. perennis perennis*, without water-soluble low molecular weight compounds, were orally administered to male mice of the ICR-CD1 strain (120 mg/Kg/day) for 30 days and were able to protect the immune and reproductive systems against toxic chemicals inducers of oxidation [99]. Purification of such polysaccharides was considered a fundamental step, since the administration of low molecular weight compounds resulted in small foci of inflammation on the animal’s testis and epididymis [99].

### 8.2. Antiviral Properties

The 70% methanol extract from *S. fruticosa* and its isolated flavonol glycosides (rhamnazin 3-*O*-2G-rhamnorutinoside or rhamnazin 3-*O*-(2′′,6′′-*O*-α-di-rhamnosyl)-β-glucoside, rhamnazin 3-O-rutinoside, rhamnazin 3-*O*-(6′′-*O*-α-rhamnosyl)-β-galactoside, isorhamnetin 3-*O*-(6′′-*O*-α-rhamnosyl)-β-galactoside, isorhamnetin 3-*O*-(2′′,6′′-*O*-α-di-rhamnosyl)-β-galactoside and allantoin, Table 5) were tested in vitro for their HCV protease activity [88]. The crude extract displayed antiviral properties, with an IC_50_ value of 10.5 μg/mL, and rhamnazin tri-glycoside had the highest HCV protease inhibitory (IC_50_ = 8.9 μM), when compared to the positive control (hepatitis virus C NS3 protease inhibitor 2, IC_50_ = 1.5 μM) [88]. Flavonol glycosides display several pharmacological activities, but there is limited information regarding the inhibition of HCV PR by flavonoids, as for example quercetin with described with HCV NS3 PR inhibition activity [109].

## 9. Propagation Systems

### 9.1. Cultivation

Data regarding the cultivation of *Sarcocornia* species can be found in Table 7. Even though *Sarcocornia* spp. are halophytic, their seeds, similarly to most halophytes, are sensitive to salinity and germinate best in freshwater. Working with *S. fructicosa* ecotypes collected in Israel, Ventura et al. [12] observed that while increasing salinity decreased the germination rate, it should be feasible to use up to 75% seawater for germination if additional seeds are available to compensate for the reduced germination rate (~40% under experimental conditions). At 25% of sea-water, germination rates reached ~90% of freshwater controls [12].

Opportunities for halophyte cultivation exist wherever saline soil conditions are found. One advantage of *Sarcocornia* is that it can be cultivated under a wide range of salinities. Ventura et al. [12] investigated ecotype biomass accumulation response to different salinity levels and observed that *Sarcocornia* biomass accumulation was not significantly affected by salinity level but differed greatly between ecotypes. This difference highlights the importance of ecotype screening for cultivation candidates.

*Sarcocornia* presents a major advantage over its “counterpart” genus, *Salicornia*: it is perennial, and therefore can be harvested year-round. Under a frequent harvest regime there will be no floral induction and the plant will not enter the reproductive phase throughout an entire, yearly cultivation cycle. This allows producers to maintain non-seasonal supply. Under natural light conditions, and without harvesting however, *Sarcocornia* will enter the reproductive phase and, in the Mediterranean area, flower in late autumn [23]. When growing plants for vegetable production only the fresh and tender parts of the plant are acceptable [8]. Using this criterion, Ventura et al. [23] developed a harvesting regime based on repeated harvests at two-, three- or four-week intervals, depending on crop growth. This technique requires that, when plants have reached 10–15 cm height, there is an initial cut, leaving approximately 5 cm of shoot above the soil surface, creating a ‘cutting table’. New shoots grow back rapidly and evenly, allowing for repeated harvesting when they attain marketable food grade height.

*Sarcocornia* has been grown successfully in Israel for more than 20 years [8], usually in greenhouses or net tunnels on 0.5–1 ha plots. Ramat Hanegev and Dead Sea farms are situated on deep free-draining dune sands where saline leachate from drip irrigation will not further contaminate the brackish water aquifers. In such areas, the salinity of the irrigation water can be as high as 10 dS/m [110]. Other cultivation methods are available in areas with other conditions, such as constructed wetlands irrigated with sea water; soilless systems utilizing sand, perlite or coir, and plastic sheet lined troughs where polystyrene rafts hold the growing crop in saline water or even briny discharge from shrimp culture ponds. The use of saline effluents that would otherwise be a disposal challenge is currently gaining much interest. For example, the use of briny reject water from reverse osmosis desalination to irrigate *Sarcocornia* should be examined and proven.

*Sarcocornia fruticosa* in a constructed wetland might be an efficient biofilter to remove organics and nitrogen of high salinity effluents from the tannery industry [111] and fishpond effluents [112]. It was found that the income generated from selling *Sarcocornia* as an agricultural crop, together with savings on water treatment and potential fines, contributes to the system's economic viability. Pinheiro and colleagues [113,114] further demonstrated the usefulness of plant-animal production systems by growing *S. ambigua* and white shrimp in a recirculating system. In addition to increasing the efficiency of nitrogen assimilation and thus reducing waste, it was possible to produce 2 kg of plant biomass per kg of shrimp. This system was not designed for commercial production but could easily be adapted for such a purpose allowing for increased revenue from the plant-shrimp aquaponic set-up. Other opportunities for cultivation lie in the use of marine algae as compost. Seaweed tend to accumulate on beaches, rendering them unusable for tourism with a negative economic effect. This seaweed is generally treated as waste and disposed of as such. It can, however, be composted and, regardless of the high salt content, be used to grow halophytes such as *S. perennis* [115]. In this way, an otherwise waste material can be effectively and efficiently re-used to provide additional revenue venues.

### 9.2. In Vitro Culture Techniques

*In vitro* culture is the aseptic culture of plants or plant organs/tissues (e.g., embryos, cells) under aseptic and controlled environmental conditions (e.g., culture medium, temperature). Micropropagation is one of the existing commercial applications of in vitro plant tissue culture tools and has several advantages over conventional methods of plant propagation, such as allowing for a faster and mass multiplication of plants with the same phenotypic and genetic characteristics, in a limited space, to obtain disease-free specimens, and is particularly relevant for species difficult or impossible to propagate by conventional methods [116].

The exploitation of *Sarcocornia* species relies on their commercial cultivation. Still, producers generally struggle to produce their seedlings or acquire them in the market. The lack of suitable seedlings in adequate quantities hampers their capacity to produce sufficient biomass with the desired quality [117]. Plant tissue culture could provide such plant material, is considered an essential tool in modern agriculture, and has been used for the massive propagation of different species with high commercial values, including strawberries [118], avocado [119] and blueberries [118]. We could only find two published reports on the *in vitro* propagation of *Sarcocornia* species. The *in vitro* micropropagation of *S. fruticosa* was accomplished from explants with three nodes, obtained from seeds germinated in solidified H&A medium [25]. Doubling the concentration of the basal culture medium and supplementing the culture media with 100 mg/L of casein hydrolysate plus 150 mg/L of glutamine and with 100 mg/L casein hydrolysate plus vitamins resulted in lengthier plantlets and enhanced multiplication rates [25]. The morphogenetic response of different explants in the in vitro culture of *S. ambigua* was evaluated by Gonçalves and Zaffari [117]. The initial explants used were young and adult nodal segments from plants from the wild, divided into apical, median, and basal regions. The highest percentage of aseptic cultures was obtained from the explants of apical and basal nodal segments pre-disinfected with kasugamycin followed by 70% ethanol and 1% calcium hypochlorite (CaClO). Supplementation of Murashige and Skoog liquid culture medium with 0.5 mg/L of naphthaleneacetic acid (NAA); 1.0 mg/L 6-benzylaminopurine (BAP) and 20 g/L sodium chloride (NaCl) allowed for a higher budding rate, while the use of natural marsh substrate in the culture medium permitted to maintain the green explants longer [117].

## 10. Conclusive Remarks and Future Perspectives

Species in the *Sarcocornia* genus present an adequate nutritional profile for human consumption, similar or even better than their counterparts in the *Salicornia* genus and other widely consumed fresh vegetables, including lettuce and spinach. *Sarcocornia* contains high levels of bioactive components, which are highly procured at the industrial level, such as polyphenolics and antioxidant vitamins, and exhibit functional biological properties, such as antioxidants. In addition, *Sarcocornia* is perennial and highly tolerant to soil and water salinity. Therefore, it can be cultivated year-round under saline agriculture, which is especially relevant under climate change, soil and water salinization and shortage of freshwater for agriculture purposes. Studies have demonstrated the economic viability of *Sarcocornia* cultivation. Future studies should optimise the saline sustainable cultivation of *Sarcocornia* species, aiming at its full commercial exploitation while maintaining or improving its nutritional and functional properties. For this purpose, the authors of this review paper are involved in the research project SaltyCrops (Exploring *Sarcocornia* as a halophyte biofilter for mariculture effluents and a source for lucrative foods and speciality biochemicals; bilateral project, Portugal/Israel, PT-IL/0003/2019) where the cultivation of different *Sarcocornia* ecotypes is being optimized, in an integrated multitrophic system (IMTA), in Portugal, combining the plant cultivation in aquaponics with the aquaculture of commercial fish, in outdoor tanks, and constructed wetlands, in Israel. Efforts should also be aimed at optimising in vitro micropropagation protocols for elite ecotypes and the biotechnological valorization of *Sarcocornia* by isolating and identifying other bioactive metabolites that they may contain.

## Figures and Tables

**Table 1:** Proximate composition of Sarcocornia species. Values are expressed as a percentage (%) and as calories per 100 g (energy) in dry weight (dw).

Species	Origin		Moisture	Crude Fat	Ash	Fibre	Energy	Crude Protein	Ref.
	Country	Source							
*S. perennis perennis*	Portugal	Wild	85.8	2.25	23.3	20.8 (NDF)	nd	6.90	[13]
*S. perennis alpini*	Portugal	Wild	84	1.20	30.7	34.1 (NDF)	nd	8.10	[13]
*S. ambigua*	Brasil		88	0.12–0.16	24.9–1.8	17.2–23.2 (IFL)	nd	16.2–18.02	[36]
*S. fruticosa*	Spain	Wild	84.1	4.45	28.9	22.0	nd	9.25	[36]
	Portugal	Wild	87	4.41	37.3	16.6	nd	9.55	[36]
	Portugal	Cultivated	92	5.60	43.3	9.26	nd	12.6	[36]
*S. neei*	Chile	Wild	89	1.08	35.8	9.98 (CF)	222.5	13.5	[35]
	Chile	Cultivated	86	1.07	35.7	16.9 (CF)	194.8	6.98	[35]

NDF, neutral detergent fiber; IFL, insoluble fiber levels; CF, crude fiber; nd, not determined.

**Table 2:** Mineral composition of *Sarcocornia* species. Values are expressed as mg per g (dry weight, dw, or fresh weight, fw).

Species/Location	*S. perennis perennis*	*S. perennis alpini*	*S. perennis perennis*	*S. ambigua*	*S. ambigua*	* S. fruticosa *			* S. neii *	
	Portugal (Wild)	Portugal (Wild)	Portugal (Wild)	Brazil	Brazil	Spain (Wild)	Portugal (Wild)	Cultivated	Wild	Cultivated
Macroelements										
Sodium (Na)	64.1 dw	64.3 dw	160 dw	10.19–16.5 fw	na	243 dw	253 dw	297 dw	17.6 fw	13.6 fw
Calcium (Ca)	2.34 dw	2.63 dw	3.10 dw	0.53–0.54 fw	2.6–4.0 dw	24.7 dw	21.3 dw	17.8 dw	6.3 fw	5.3 fw
Potassium (K)	13.9 dw	10.3 dw	11.1 dw	1.81–2.90 fw	19–24 dw	62.4 dw	49.6 dw	87.5 dw	20.3 fw	15.5 fw
Magnesium (Mg)	6.73 dw	7.03 dw	4.98 dw	0.92–1.30 fw	8.6–14 dw	59.2 dw	28.7 dw	17.4 dw	11.3 fw	8.2 fw
Phosphorus (P)	na	na	2.2 dw	na	na	na	na	na	1.8 fw	1.6 fw
Microelements										
Iron (Fe)	0.82 dw	1.28 dw	0.35 dw	na	na	1.69 dw	0.68 dw	0.68 dw	0.33 dw	0.276 dw
Selenium (Se)	na	na	na	na	0.002–0.0025 dw	na	na	na	na	na
Manganese (Mn)	0.03 dw	0.06 dw	–	na	0.019–0.052 dw	0.155 dw	0.03 dw	0.04 dw	0.1 fw	0.04 fw
Copper (Cu)	na	na	0.011 dw	na	0.004–0.013 dw	0.0243 dw	0.0168 dw	0.0161 dw	0.0172 fw	0.01 fw
Zinc (Zn)	0.016 dw	0.025 dw	0.021 dw	na	0.027–0.041 dw	0.0589 dw	0.0294 dw	0.0433 dw	0.028 fw	0.04 fw
Cadmium (Cd)	nd	0.00019 dw	0.0004 dw	na	na	nd	nd	nd	0.00004 fw	0.00032 fw
Nickel (Ni)	0.00191 dw	0.00164 dw	na	na	0.200–0.003 dw	0.0201 dw	0.00348 dw	0.0139 dw	na	na
Lead (Pb)	0.00045 dw	0.00131 dw	0.001 dw	na	0.0004–0.0008 dw	nd	nd	nd	0.0123 fw	0.00972 fw
Chromium (Cr)	0.00467 dw	0.00492 dw	na	na	0.0013–0.002 dw	0.0569 dw	0.0084 dw	0.029 dw	na	na
References	[13]	[13]	[56]	[34]	[5]	[36]			[35]	

na, not analysed; nd, not detected.

**Table 3:** Fatty acids composition (% of total fatty acids) of *Sarcocornia* species.

		Species and Organ					
Fatty Acid		*S. perennis perennis*	*S. perennis alpini*	*S. ambigua * ^a^	*S. ambigua * ^b^	* S. ambigua *	* S. ambigua *
Common Name	Carbon Length	Leaves	Leaves	Leaf and Steam	Leaf and Steam	Seed Oil	Fertile Shoots (with Seeds)
Lauric acid	C12:0	0.39	0.61	0.45	0.45	-	-
Myristic acid	C14:0	1.3	1.95	0.59	0.58	-	2.1
Pentadecyclic acid	C15:0	0.3	0.64	-	-	-	-
Palmitic acid	C16:0	17.9	21.2	13.2	13.65	20.4	19.8
Palmitoleic acid	C16:1	1.67	3.78	0.65	0.67	1.4	0.8
Margaric acid	C17:0	0.56	0.62	-	-	-	-
Stearic acid	C18:0	2.04	3.67	1.7	1.7	4.5	4.6
7-cis-octadecenoic acid	C18:1n-7	-	-	-	-	0.6	-
Oleic acid	C18:1n-9	0.81	4.69	3.84	3.92	18.5	18.3
Linoleic acid (LA)	C18:2n-6	18.7	17.5	16.57	16.62	42.0	21.4
α-Linolenic acid (ALA)	C18:3n-3	21.1	19.3	44.04	44.7	4.0	-
Stearidonic acid	C18:4	-	-	-	-	1.5	-
Arachidic acid	C20:0	5.74	4.45	0.79	0.79	-	3.0
Eicosenoic acid	C20:1n-9	nd	nd	-	-	-	-
Heneicosylic acid	C21:0	1.14	3.2	-	-	-	1.4
Behenic acid	C22:0	11.1	8.27	1.08	1.09	-	7.1
2-Hydroxydocosanoic acid	OH-C22:0	-	-	-	-	-	1.4
Erucic acid	C22:1n-9	nd	2.26	-	-	-	-
Tricosylic acid	C23:0	7.9	1.33	-	-	-	2.2
Lignoceric acid	C24:0	6.75	6.51	-	-	-	5.3
Cerotic acid	C26:0	2.56	nd	-	-	-	2.6
Montanic acid	C28:0	nd	nd	-	-	-	4.2
Melissic acid	C30:0	-	-	-	-	-	0.20
Total SFA		57.7	52.4	17.82	18.27	24.9	53.9
Total MUFA		2.47	10.7	4.49	4.59	20.5	19.1
Total PUFA		39.9	36.9	60.61	61.31	47.5	21.4
References		[13]	[13]	[34]	[34]	[26]	[14]

^a^ Region A: Natural tideland near the coast of Brasil; ^b^ Region B: From an experimental crop irrigated with seawater and fertilized with sludge of shrimp farm in Brasil; nd, not detected; SFA: Saturated fatty acids; MUFA: monounsaturated fatty acids; PUFA: polyunsaturated fatty acids. Nd, not detected; - not analysed.

**Table 4:** Vitamin contents of *Sarcocornia* species. Values are expressed as mg per g (dry weight, dw, or fresh weight, fw).

Species	Origin		Vitamin E		Vitamin C	Vitamin B6	Ref.
	Country	Source	α-Tocopherol	γ-Tocopherol			
*S. perennis perennis*	Portugal	Wild	0.0111 dw	0.0009 dw	na	na	[13]
*S. perennis alpini*	Portugal	Wild	0.0201 dw	0.0096 dw	na	na	[13]
*S. ambigua*	Brasil	Wild	na	na	0.237–0.654 fw	na	[34]
*S. fruticosa*	Spain	Wild	0.176 dw	na	0.0038 fw	0.0981 dw	[36]
	Portugal	Wild	0.934 dw	na	0.0042 fw	0.0667 dw	[36]
	Portugal	Cultivated	nd	na	0.0076 fw	0.0758 dw	[36]
*S. neei*	Chile	Wild	na	na	0.55 dw	na	[35]
	Chile	Cultivated	na	na	0.9 dw	na	[35]

na, not analysed.

**Table 5:** Phenolic compounds identified in Sarcocornia species.

Chemical Structure	Name	Species	References
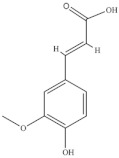	ferulic acid	* S. ambigua *	[34]
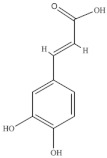	caffeic acid		
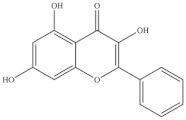	galangin		
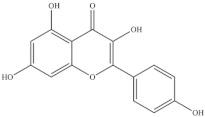	kaempferol	* S. ambigua *	[72]
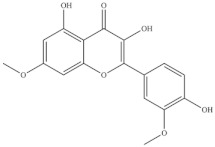	quercetin		
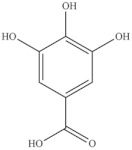	gallic acid		
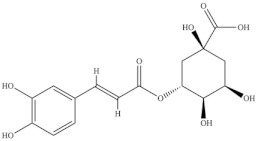	chlorogenic acid	* S. fruticosa *	[3]
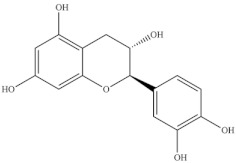	catechin hydrate		
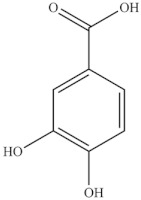	3,4-dihydroxybenzoic acid		
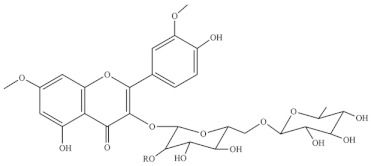	3-O-2′^G^′-rhamnorutinoside	* S. fruticosa *	[88]
	rhamnazin 3-O-rutinoside		
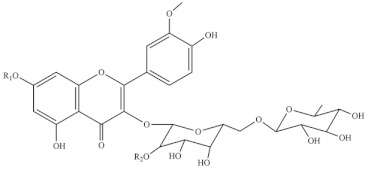	rhamnazin 3-O-(6′′-O-α-rhamnosyl)-β-galactoside		
	isorhamnetin 3-O-(6′′- O-α-rhamnosyl)-β-galactoside		
	isorhamnetin 3-O-(2′′,6′′-O-α-di- rhamnosyl)-β-galactoside		
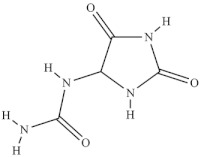	allantoin		

**Table 6:** Bioactive properties of *Sarcocornia* species.

Species/Bioactivity	Plant Organ	Extract/Compounds	Assay	Results	Ref.
Antioxidant properties					
* S. ambigua *	Vegetative and reproductive segments	Water, 80% ethanol, 80% acidified aqueous methanol and methanol/ethanol/water (40/40/20)	ABTS	3.4–4.9 mmol TEAC/100 g FW	[72]
* S. perennis alpini *	Shoots	Ethanol	DPPH	IC_50_ = 11.5 mg/mL	[13]
* S. perennis alpini *	Shoots	Ethanol	FRAP	IC_50_ = 6.55 mg/mL	[13]
* S. perennis perennis *	Shoots	Ethanol	DPPH	IC_50_ = 8.04 mg/mL,	[13]
* S. perennis perennis *	Shoots	Ethanol	FRAP	IC_50_ = 4,57 mg/mL	[13]
* S. perennis *	Aerial organs	Methanol	DPPH	IC_50_ = 0.43 mg/mL	[15]
S. *ambigua*	Aerial organs	Methanol	DPPH	135.83 μmol TEAC/100 g	[34]
			FRAP	170.14 μmol Fe^+2^/100 g	[34]
* S. perennis perennis *	Aerial organs	Ethanol:water (75:25)	TEAC	2.54 mM Trolox/100 g	[56]
			ORAC	8.84 mM Trolox/100 g	
* S. fruticosa *	Aerial organs	Methanol	DPPH	IC_50_ = 4.3 μM	[88]
* S. perennis *	Aerial organs	Methanol crude extract Methanol fraction 80 and 60%	Lead (Pb)-induced oxidative damages in HEK293 kidney cells	Decreased superoxide release	[15]
* S. perennis *	Aerial organs	Water	Pb toxicity in rats	Increase in the activity of GSH, SOD, CAT and GPx	[83]
Antiviral properties					
* S. fruticosa *	leaves	Flavonol rhamnazin tri-glycoside isolated from a water/methanol extract	In vitro HCV inhibition	IC_50_ = 8.9 μM	[88]

SOD: superoxide dismutase; CAT: catalase; GPx: glutathione-peroxidase; Hepatitis C Virus (HCV).

**Table 7:** Sites and modes of cultivation of *Sarcocornia* species.

Species	System	Country	Other Species in the System	Harvest Regime	Evaluated Parameters	Ref.
* S. ambigua *	Aquaponics with biofloc technology using NFT system	Brasil	* Litopenaeus vannamei *	73 days	Plant production, growth rate of shrimps, total N in shrimp, plants and feed, antioxidant activity, total phenolics in plants	[113]
* S. ambigua *	Aquaponics with NFT system evaluated at 8, 16, 24, and 32 psu	Brasil	* L. vannamei *	57 days	Plant production, growth rate of shrimps, N and P use efficiency, antioxidant activity, total phenolics in plants	[114]
* S. ambigua *	Plots irrigated with saline effluent from shrimp tank	Brasil	*L. vannamei*	6 months	Phenolics, chlorophylls, L-ascorbic acid, antioxidant capacity of mature shoots	[72]
* S. fructicosa *	Cultivation in pots with perlite irrigated with different seawater concentrations	Israel	* Salicornia persica *	Cycle of three weeks	Yield production, nutritional value, harvest regime	[12]
* S. fructicosa *	Constructed wetlands at a leather company	Portugal	* Arundo donax *	370 days	COD, BOD_5_, TSS, TDS, TKN, NO_3_^−^, NH_4_^+^, TP, Cl^−^, plant propagation and growth, uptake of N and P	[111]
* S. perennis *	Cultivation in pots with different proportions of seaweed compost and perlite.	Argentina	*Undaria pinnatifida*, *Ulva* sp., *Ceramium* sp., *Dyctiota dichotoma*, *Corallina* sp., *Codium* sp., *Gracilaria gracilis*	42 days	Plant growth, water content, C and N content in plants	[115]

NFT: nutrient film technique; COD: chemical oxygen demand; BOD_5_: biochemical oxygen demand; TSS: total suspended solids; TDS: total dissolved solids; TKN: Total Kjeldahl Nitrogen; NO_3_^−^: nitrate nitrogen; NH_4_^+^: ammonium nitrogen; TP: total phosphorus; Cl^−^: chloride.

## Data Availability

Data is contained within the article.
